# Gut microbiota helps identify clinical subtypes of Parkinson’s disease

**DOI:** 10.1186/s40779-024-00545-4

**Published:** 2024-07-01

**Authors:** Jing-Yi Wang, Rui Xie, Yun Feng, Min-Na Zhang, Le He, Bo Yang, Hong-Gang Wang, Xiao-Zhong Yang

**Affiliations:** 1 Department of Gastroenterology, the Affiliated Huai’an No.1 People’s Hospital of Nanjing Medical University, Huai’an, 223301 Jiangsu China; 2 Department of Radiology, the Affiliated Huai’an No.1 People’s Hospital of Nanjing Medical University, Huai’an, 223301 Jiangsu China

**Keywords:** Parkinson’s disease, Gut microbiota, Functional magnetic resonance imaging

Dear Editor,

The role of the microbiota-gut-brain axis in Parkinson’s disease (PD) has garnered increasing attention [1]. Among the various empirical subtype systems of PD, motor subtype classification into tremor-dominant (TD) and postural instability and gait difficulty (PIGD) is the most commonly utilized [2]. However, the relationship between gut microbiota and clinical subtypes of PD remains unclear.

This study aimed to investigate the distinctions in gut microbiota between the two motor subtypes by obtaining fresh stool samples from patients with PD for metagenomic sequencing, along with assessing brain function using resting-state functional magnetic resonance imaging (rs-fMRI). Subsequently, differences in brain mean amplitude of low-frequency fluctuation (mALFF) values and their association with gut microbiota were analyzed.

A total of 24 patients with PD were included in the study, comprising 12 PIGD, 9 TD, and 3 indeterminate (IND) subtype patients (Additional file 1: Materials and methods). Following exclusion of the 3 IND subtype patients, no significant differences in age, sex, body mass index (BMI), Hoehn-Yahr score, Unified Parkinson’s Disease Rating Scale part III (UPDRS-III) score, Hamilton Depression Scale (HAMD) score, Hamilton Anxiety Scale (HAMA) score, drug use, duration of disease, and medical history including hypertension, diabetes, heart disease, and cerebral infarction were observed between the TD and PIGD groups (*P* > 0.05, Additional file 1: Table S1). Alpha diversity of the gut microbiota was evaluated using Chao1, Shannon, and Simpson indices, while beta diversity was assessed through principal coordinates analysis (PCoA) and nonmetric multidimensional scaling (NMDS). However, the analysis revealed no significant variations in alpha and beta diversities between the two groups (*P* > 0.05, Additional file 1: Fig. S1).

The gut microbiota was further analyzed at various taxonomic levels using a cladogram and bar plot generated by Liner discriminant analysis effect size (LEfSe). The PIGD group exhibited enrichment in *Bacteroidetes*, *Bacteroidia*, and *Bacteroidales*, while the TD group showed predominant enrichment for *Firmicutes*, *Clostridiaceae*, and *Clostridium* (Fig. 1a). At the phylum level, the proportion of *Bacteroidetes* was significantly higher in PIGD group compared with TD group (45.57% vs. 23.64%, *P* = 0.003, Fig. 1b).

**Fig. 1:**
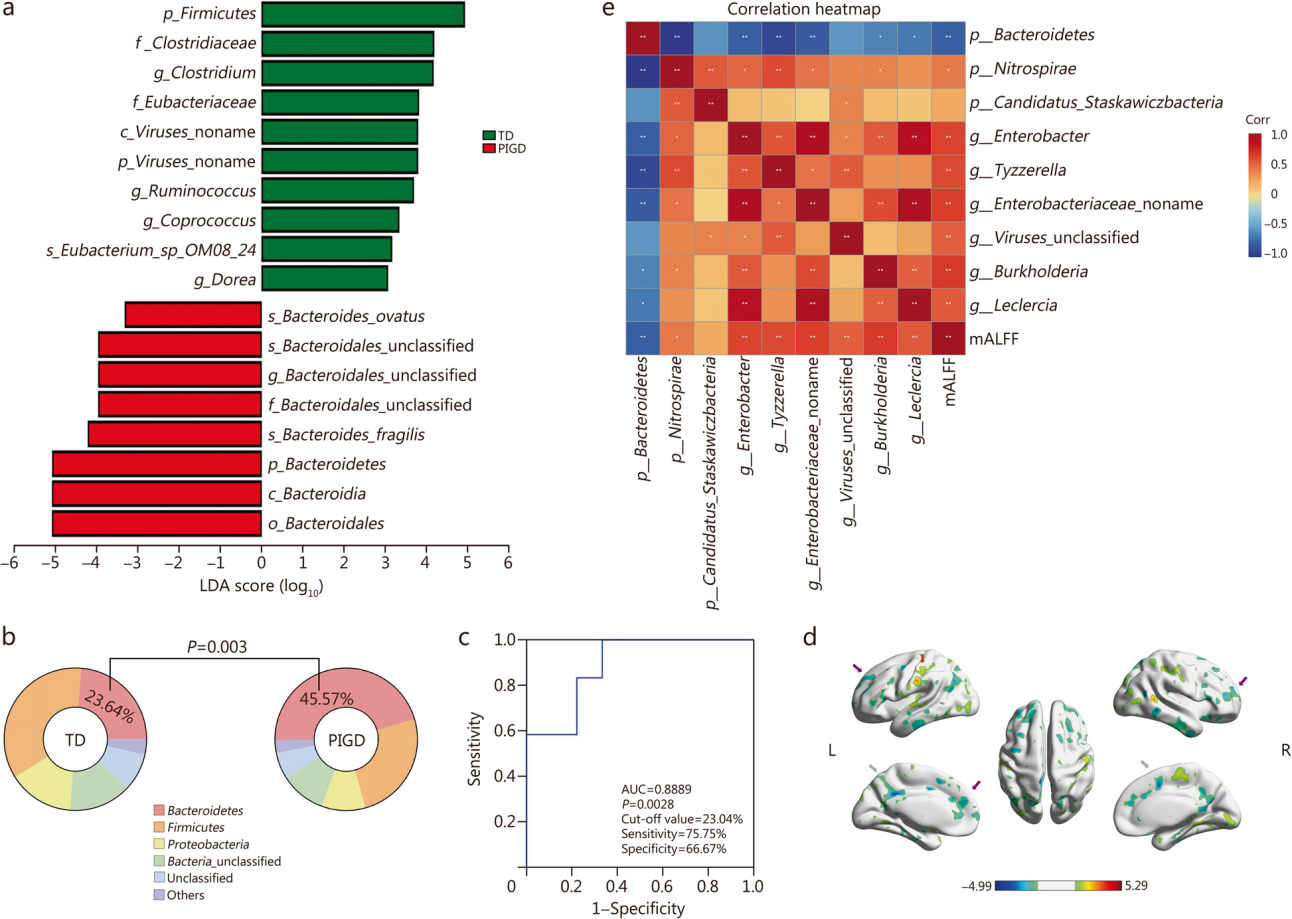
Microbiota-gut-brain axis in PD. **a** Liner discriminant analysis effect size (LEfSe) analysis and Cladogram generated by LEfSe analysis. **b** Different proportion of phylum and statistical difference between the TD and PIGD subtypes (*P* = 0.003). **c** ROC curve. **d** Significant brain changes between the TD and PIGD subtypes (gray arrows: bilateral median cingulate and paracingulate gyri; purple arrows: precuneus; brown arrow: parietal lobes). **e** Spearman analysis was performed for correlation analysis. PD Parkinson’s disease, TD tremor-dominant, PIGD postural instability and gait difficulty, mALFF mean amplitude of low-frequency fluctuation, Corr correlation coefficient

A binary logistic regression model was developed using the proportion of *Bacteroidetes* to predict the motor subtypes of patients with PD. The ROC curve analysis yielded an AUC of 0.8889 (*P* = 0.0028), with a cut-off value of 23.04%, sensitivity of 75.75%, and specificity of 66.67% (Fig. 1c). Additionally, at the genus level, PIGD group exhibited significantly lower abundance of *Clostridium*, *Ruminococcus*, *Coprococcus*, *Dorea*, *Enterobacter*, *Anaerotignum*, *Tyzzerella*, *Bacillus*, and *Evtepia* compared with TD group (*P* < 0.05, Additional file 1: Fig. S2).

Kyoto Encyclopedia of Genes and Genomes (KEGG) pathway enrichment analysis was conducted to explore differences in gene abundance between TD and PIGD patients, revealing significant enrichment in various categories including environmental information processing, human diseases, cellular processes, genetic information processing, and metabolism. Notably, the metabolic pathway emerged as the most significantly enriched pathway in KEGG pathway classification (Additional file 1: Fig. S3).

Functional brain MRI data from these patients with PD were utilized for brain mALFF analysis in this study. In comparison with TD group, individuals in PIGD group exhibited reduced mALFF signals in the bilateral median cingulate and paracingulate gyri, precuneus, and parietal lobes (Fig. 1d). The right median cingulate and paracingulate gyri were identified as the key brain regions, and the local mALFF values were collected from each participant. Subsequently, Spearman’s correlation analysis was conducted between the extracted mALFF values and various significantly distinct gut microbiotas (Fig. 1e). The findings revealed a positive correlation between the abundance of *Enterobacter*, *Tyzzerella*, *Burkholderia*, and *Leclercia* and the mALFF values (*P* < 0.05). Conversely, the abundance of *Bacteroidetes* showed a negative correlation with the mALFF values (*P* < 0.05).

Our research contributes valuable insights into the microbiota-gut-brain axis and underscores the potential relationship between gut microbiota and motor subtypes in PD. These findings lay the groundwork for future investigations into the pathogenesis of distinct motor subtypes. Despite being a small-scale study, we have innovatively established a connection between gut microbiota and brain function MRI, offering potential for future regulation of gut-directed therapies in PD.

### Supplementary Information

**Additional file 1:** **Materials and Methods. Table S1** Characteristics of different motor subtypes (TD and PIGD) in PD patients. **Fig. S1** Alpha diversity and beta diversity of the gut microbiota. **Fig. S2** Relative abundance of discriminative gut microbiota at the genus level (*P *< 0.05). **Fig. S3** Kyoto Encyclopedia of Genes and Genomes (KEGG) pathway enrichment analysis.

## Data Availability

The data presented in this study are available upon reasonable request from the corresponding author.

## Abbreviations

AUC: Area under the receiver operating characteristic curve
HAMD: Hamilton Depression Scale
HAMA: Hamilton Anxiety Scale
IND: Indeterminate
KEGG: Kyoto Encyclopedia of Genes and Genomes
LEfSe: Liner discriminant analysis effect size
mALFF: Mean amplitude of low-frequency fluctuation
NMDS: Nonmetric multidimensional scaling
PD: Parkinson’s disease
PIGD: Postural instability and gait difficulty
PCoA: Principal coordinates analysis
rs-fMRI: Resting-state functional magnetic resonance imaging
TD: Tremor-dominant
UPDRS: Unified Parkinson’s Disease Rating Scale

## References

[1] Stopińska K, Radziwoń-Zaleska M, Domitrz I (2021). The microbiota-gut-brain axis as a key to neuropsychiatric disorders: a mini review. J Clin Med.

[2] Mestre TA, Fereshtehnejad SM, Berg D, Bohnen NI, Dujardin K, Erro R (2021). Parkinson's disease subtypes: critical appraisal and recommendations. J Parkinsons Dis.

