# Supplementary material for: Gut microbiota helps identify clinical subtypes of Parkinson’s disease
Source: Mil Med Res. 2024 Jul 1;11:42. doi: 10.1186/s40779-024-00545-4 (PMC11218113; doi:10.1186/s40779-024-00545-4)
Supplement: Supplementary file 1 — Additional file 1: Materials and Methods. Table S1 Characteristics of different motor subtypes (TD and PIGD) in PD patients. Fig. S1 Alpha diversity and beta diversity of the gut microbiota. Fig. S2 Relative abundance of discriminative gut microbiota at the genus level (P < 0.05). Fig. S3 Kyoto Encyclopedia of Genes and Genomes (KEGG) pathway enrichment analysis. [file 40779_2024_545_MOESM1_ESM.pdf]

Materials and Methods

Participants

A total of 24 PD patients from the Department of Neurology at the Affiliated Huai'an No.1 People's Hospital of Nanjing Medical University were recruited to participate in the study between January to December 2022. All participants were examined by a movement disorder specialist and diagnosed according to the International Parkinson and Movement Disorder Society (MDS) clinical diagnostic criteria [1]. Exclusion criteria comprised the following: 1) atypical or secondary parkinsonism; 2) chronic gastrointestinal disease; 3) serious chronic diseases with gastrointestinal involvement such as renal failure, heart failure and diabetes; 4) a history of antibiotic use in the last month; 5) difficulty in completing the clinical evaluation.

The patients were divided into three groups, including 12 with the postural instability and gait difficulty (PIGD), 9 with the tremor-dominant (TD), and 3 with the indeterminate (IND). Among them, 3 patients with PD of the IND subtype were excluded from subsequent studies. During the study period, patients continued to receive stable doses of PD. This study was approved by the Medical Ethics Committee of Affiliated Huai'an No.1 People's Hospital of Nanjing Medical University (KY-2022-004-01). Written informed consent was obtained from all individuals participating in the study prior to their involvement in the experiments.

Motor subtype classification

Motor subtype was calculated based on a previously published formula using 4 items from the Unified Parkinson's Disease Rating Scale (UPDRS) part II (UPDRS-II) and 4 items from the part III (UPDRS-III). From the UPDRS-II, the items were 2.13 (falling unrelated to freezing), 2.14 (freezing when walking), 2.15 (walking), and 2.16 (tremor). From the UPDRS-III, the items were 3.20 (tremor at rest), 3.21 (action or postural tremor of hands), 3.29 (gait), and 3.30 (postural stability). The tremor-dominant (TD) score was calculated as the mean of the summation of items 2.16, 3.20, and 3.21, whereas the postural instability and gait difficulty (PIGD) score was calculated as the mean of the summation of items 2.13, 2.14, 2.15, 3.29, and 3.30. The motor subtype was defined according to the following quotient. (1) TD: TD score/PIGD score ≥ 1.5; (2) PIGD: TD score/PIGD score ≤ 1; (3)

Indeterminate (IND): 1 < TD score/PIGD score < 1.5. All patients were assessed during the OFF state (without medication in the last 12 h), when the motor subtype was defined.

Clinical assessments

During the OFF state, the demographic characteristics of PD patients were collected, specifically age at assessment, sex, height, and weight. UPDRS-III and Hoehn-Yahr scores were used to assess motor disability and disease severity, respectively. Mood was measured using the Hamilton Depression Scale (HAMD) and Hamilton Anxiety Scale (HAMA).

Fecal sample collection

Fresh stool samples were collected from participants after waking in the morning and before medication administration. All samples were stored at –80 °C until DNA extraction.

Metagenomic sequencing of the gut microbiota

DNA from fecal samples was extracted using The E.Z.N.A.® Stool DNA Kit (D4015-02, Omega, Inc., USA) according to the manufacturer's instructions. The total DNA was eluted in 50 µl elution buffer using a modification of the procedure described by the manufacturer (Qiagen, Germany) and stored at –80 °C until measurement by LC-Bio Technologies (Hangzhou) Co., Ltd., Hang Zhou, Zhejiang Province, China. A DNA library was constructed using the TruSeq Nano DNA LT Library Preparation Kit (FC-121-4001, Illumina). DNA was fragmented using dsDNA Fragmentase (NEB, M0348S, USA) by incubating at 37 °C for 30 min. After quality control of the libraries, high-throughput sequencing was performed using NovaSeq6000 in the sequencing mode of PE150. Raw data obtained from sequencing were subjected to further analysis. First, sequencing adapters were removed from the sequencing reads using the Cutadapt (v1.9). Second, low-quality reads were trimmed using fqtrim (v0.94), using a sliding-window algorithm. Reads were then aligned to the host genome using Bowtie2 (v2.2.0) to remove host contamination. Once quality-filtered reads were obtained, they were assembled de novo to construct the metagenome for each sample using the

MEGAHIT (v1.2.9). All coding regions (CDS) of the metagenomic contigs were predicted using MetaGeneMark (v3.26). The CDS sequences of all samples were clustered using CD-HIT v4.6.1, to obtain unigenes. Unigene abundance for a certain sample was estimated by transcripts per million (TPM) based on the number of aligned reads Bybowtie2 (v2.2.0). Unigenes were obtained after filtering for low abundance expression, and unigenes were compared with the NR_mate library to obtain species annotation information by DIAMOND (v0.9.14). Unigenes were compared with Kyoto Encyclopedia of Genes and Genomes (KEGG) databases for functional annotation. Alpha and beta diversity were determined using QIIME2, and pictures were drawn using R (v3.5.2). Among them, alpha diversity of the gut microbiota was evaluated using Chao1, Shannon, and Simpson indices, while beta diversity was generated based on Bray-Curtis distances for both principal coordinates analysis (PCoA) and nonmetric multidimensional scaling (NMDS) analysis. Linear discriminant analysis and effect size (LEfSe) analyses were performed using the LEfSe tool (http://huttenhower.sph.harvard.edu/lefse/).

Magnetic resonance imaging (MRI) data acquisition, processing and analysis

A 32-channel 3.0-T MRI scanner (Philips, Ingenia 3.0 CX, The Netherlands) was used for MR images, and the patient was placed in a relaxed state by sitting quietly for 15 min before scanning, with sound-deadening soft earplugs and a sponge slope pad on the leg of the elderly patient. The resting-state functional magnetic resonance imaging (rs-fMRI) parameters were as follows: repetition time (TR) = 2000 ms, echo time (TE) = 30 ms, flip angle (FA) = 90°, matrix = 96 × 94, and field of view (FOV) = 230 mm × 230 mm. The scan duration was 8 min, and 250 time-points were recorded. The parameters of the 3D-T_1 images were as follows: slice thickness = 1.0 mm, TR = 6.7 ms, TE = 3.0 ms, FA = 12°, number of excitation (NEX) = 1, FOV = 240 mm × 240 mm, matrix = 256 × 256, voxel size = 1 mm × 1 mm × 1 mm. The scan sequence included routine T_2-fluid attenuated inversion recovery (FLAIR) to rule out primary brain lesions.

Image preprocessing was performed based on the Metlab2016a platform DPASF software (http://rfmri.org/DPASF). The first 10 time points of the functional images were excluded, followed by slice timing correction, realignment and head motion correction, and exclusion of subjects with

head motion > 1.5 mm, translations or rotations > 1.5°. The raw space of the subjects was estimated into standard Montreal Neurological Institute (MNI) space with normalized resampling using 3 mm × 3 mm × 3 mm for each voxel, spatial smoothing using 6 mm × 6 mm × 6 mm, filtering in the 0.01 – 0.08 Hz band, and finally multiple linear regression to analyze the effects of head motion parameters, white matter, and cerebrospinal fluid signals. The mean amplitude of low-frequency fluctuation (mALFF) was obtained from the pre-processed rs-fMRI. ALFF reflects the bold signal intensity of regional spontaneous activity. ALFF is the mean square root of voxels in the (0.01 − 0.08 Hz) frequency range. The mALFF of each voxel was normalized by the global mean ALFF within a brain mask to obtain standardized ALFF (mALFF). Differences in mALFF between the two groups were calculated using independent two-sample *t*-test with head movement parameters as covariates. Results were corrected by Gaussian random field theory (GRF) voxel *P*-value < 0.001, (cluster *P*-value < 0.05). The peak voxel MNI coordinate was selected as region of interest (ROI). The mALFF values of the peak voxel were extracted using the Rest (http://www.fil.ion.ucl.ac.uk/spm) software tool for subsequent statistical analysis.

Statistical analysis

Clinical characteristics of the participants were analyzed using the SPSS 26.0 statistical package for the Social Sciences (IBM SPSS Statistics). Fisher's exact test was used to analyze categorical data. An independent samples *t*-test was used for quantitative data with normal distribution, and the results were expressed as mean ± standard deviation (SD). The Mann-Whitney test was used for quantitative data with non-normal distribution, and the results were expressed as M (Q_1, Q_3). *P*-value (set at tails) < 0.05. Spearman correlation analysis and binary logistic regression analysis were performed using SPSS software. The area under the receiver operating characteristic (AUC) curve was evaluated to test the predictive performance of the established model. The cut-off value, sensitivity, and specificity were calculated using the Youden index.

References

1. Postuma RB, Berg D, Stern M, et al. MDS clinical diagnostic criteria for Parkinson's disease. Mov Disord. 2015;30(12):1591-601.

Table S1 Characteristics of different motor subtypes (TD and PIGD) in PD patients

**Item**	**PIGD (*n* = 12)**	**TD (*n* = 9)**	***P*-value**
Age (years, mean ± SD)	66.9 ± 6.7	61.6 ± 6.6	0.08
Sex [male, *n*(%)]	8 (66.7)	4 (44.4)	0.40
BMI (kg/m^2, mean ± SD)	23.76 ± 2.40	23.11 ± 2.32	0.54
Hoehn-Yahr score (mean ± SD)	1.92 ± 0.87	1.67 ± 0.71	0.49
UPDRS-III score (mean ± SD)	29.33 ± 10.39	31.56 ± 15.96	0.70
HAMA score [M (Q_1, Q_3)]	6 (4, 9)	14 (7, 23)	0.06
HAMD score (mean ± SD)	11.08 ± 5.93	17.11 ± 9.92	0.10
Drug use [*n*(%)]			
Oral levodopa-based therapy	12 (100.0)	9 (100.0)	1.00
Dopamine agonist	12 (100.0)	8 (88.9)	0.25
MAO-B inhibitor	2 (16.7)	0	0.21
COMT inhibitor	0	0	1.00
Amantadine	3 (25.0)	2 (22.2)	0.89
Anticholinergic	0	0	1.00
Duration of disease (years, mean ± SD)	3 (2, 7)	4 (1, 7)	0.97
Medical history [*n*(%)]			
Hypertension	4 (33.33)	2 (22.2)	0.59
Diabetes	1 (8.3)	1 (11.1)	0.83
Heart disease	0	1 (11.1)	0.25
Cerebral infarction	3 (25.0)	2 (22.2)	0.89

P-values were calculated using independent samples *t*-test, Chi-square test or Kruskal-Wallis *H*-test. *PD* Parkinson's disease, *TD* tremor-dominant, *PIGD* postural instability and gait difficulty, *BMI* body mass index, *UPDRS-III* Unified Parkinson's Disease Rating Scale part III, *HAMA* Hamilton Anxiety Scale, *HAMD* Hamilton Depression Scale, *MAO-B* monoamine oxidase B, *COMT* catechol-O-methyltransferase

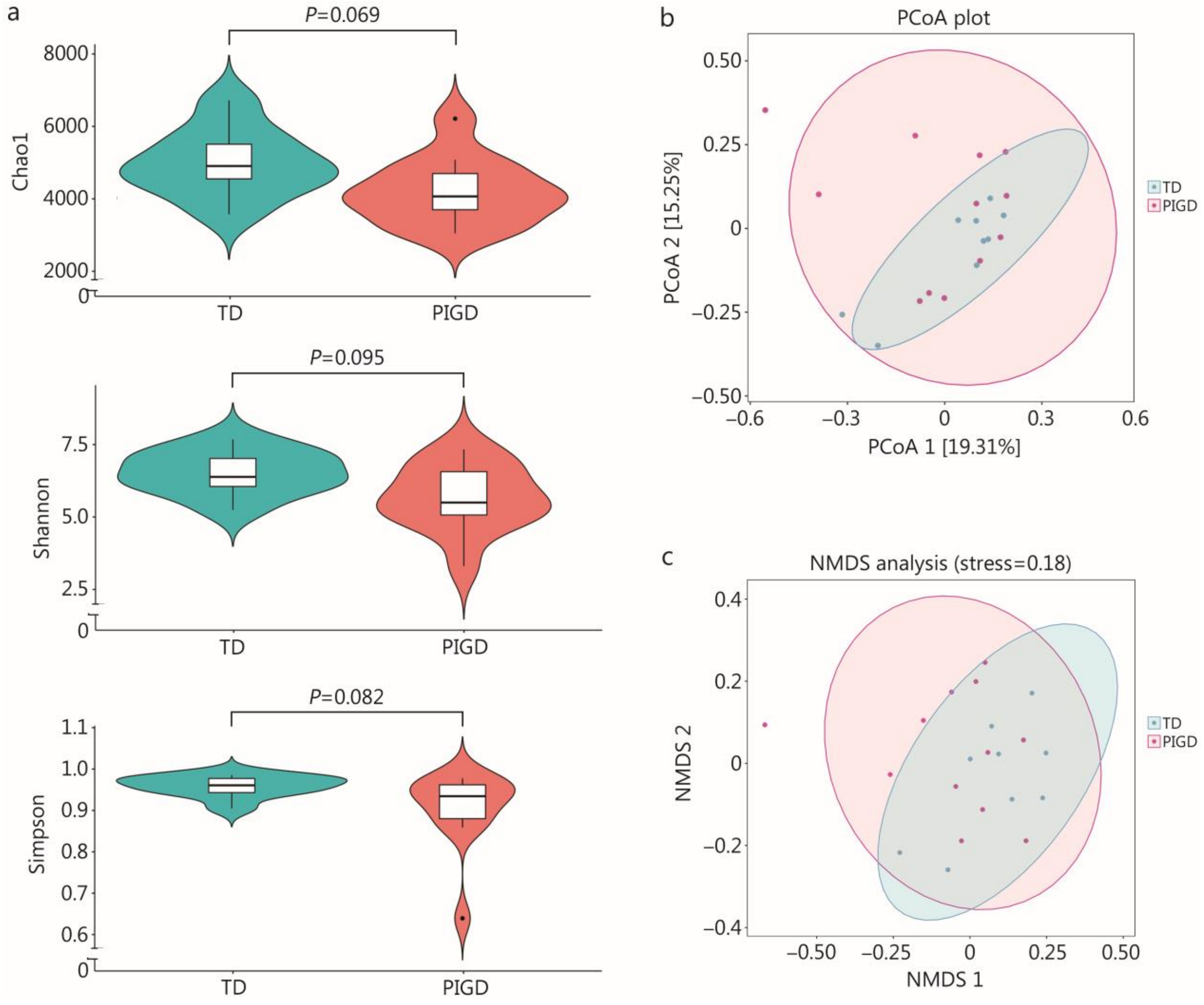

Fig. S1 Alpha diversity and beta diversity of the gut microbiota. **a** Chao1, Shannon and Simpson indices; **b** PCoA plot; **c** NMDS analysis. TD tremor-dominant, PIGD postural instability and gait difficulty, PCoA principal coordinates analysis, NMDS nonmetric multidimensional scaling

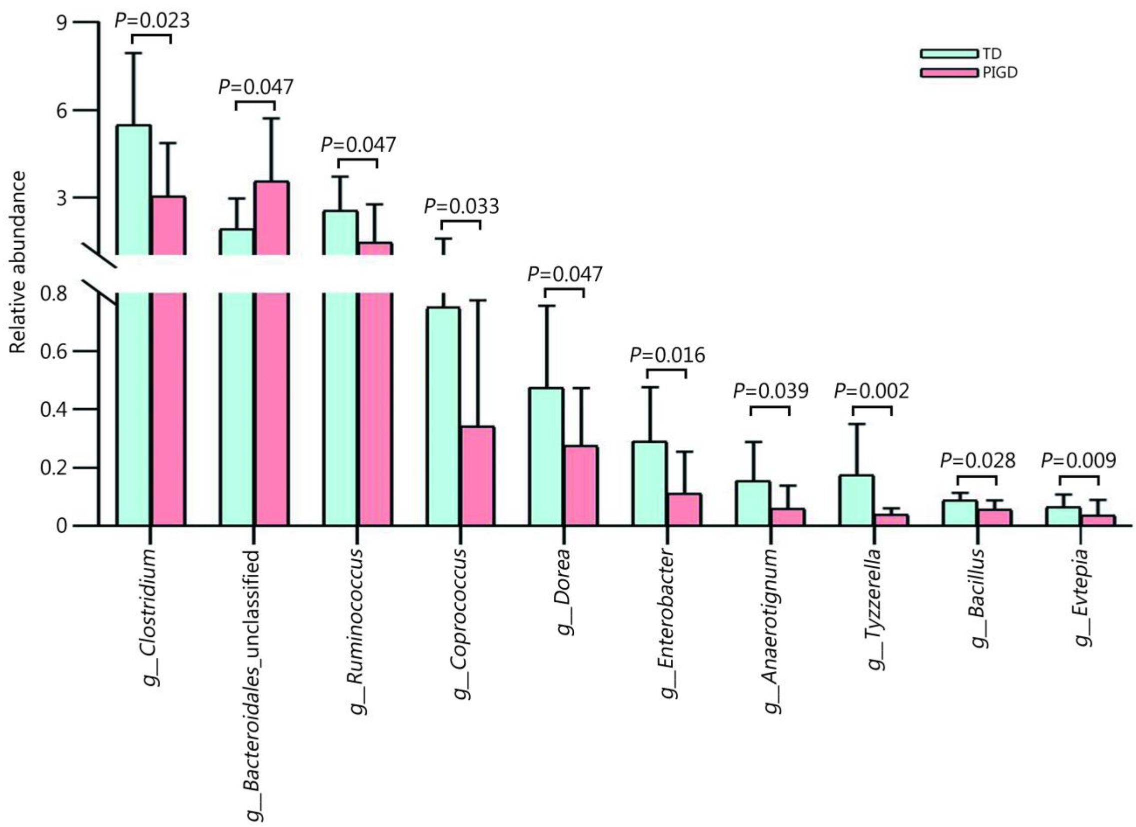

Fig. S2 Relative abundance of discriminative gut microbiota at the genus level (P < 0.05). TD tremor-dominant, PIGD postural instability and gait difficulty

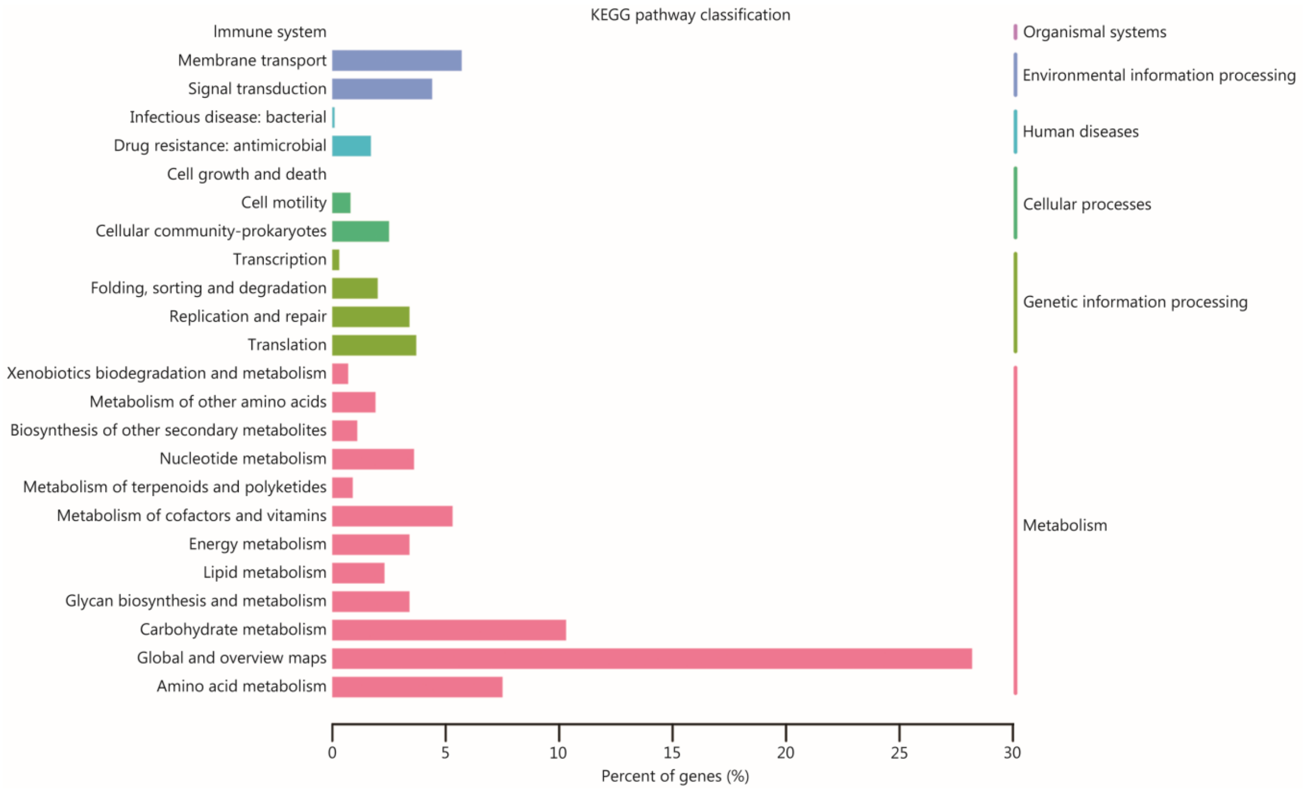

Fig. S3 Kyoto Encyclopedia of Genes and Genomes (KEGG) pathway enrichment analysis.
